# Canines and inflammatory external apical resorption in healthy maxillary lateral incisors due to occlusal trauma: when to detect the position of maxillary canines, to prevent it?

**DOI:** 10.1590/2177-6709.27.1.e22ins1

**Published:** 2022-04-11

**Authors:** Alberto CONSOLARO, Mauricio de Almeida CARDOSO, Renata Bianco CONSOLARO, Raquel Assed Bezerra SEGATO

**Affiliations:** 1 Universidade de São Paulo, Faculdade de Odontologia de Bauru (Bauru/SP, Brazil).; 2 Universidade de São Paulo, Faculdade de Odontologia de Ribeirão Preto, Programa de Pós-graduação em Odontopediatria (Ribeirão Preto/SP, Brazil).; 3 Faculdade de Medicina e Odontologia São Leopoldo Mandic, Programa de Pós-graduação em Ortodontia (Campinas/SP, Brazil).; 4 Centro Universitário de Adamantina (Adamantina/SP, Brazil).

**Keywords:** Unerupted canines, Inflammatory root resorption, Occlusal trauma

## Abstract

**Justification:**

Canines represent corners in the dental arch, and are important features in facial esthetics, as they support the upper lip, wing of the nose, and influence the nasolabial fold and the appearance of facial aging. In the laterality movements, the canines guidance coordinate the opening and closing of the teeth, saving the TMJ from sudden movements.

**Discussion:**

As a result of the lack of eruption or the inadequate positioning of the maxillary canine, the loss of the laterality guide may occur, which will then occur in the maxillary lateral incisor, inducing lesions of “occlusal trauma”, such as inflammatory root resorption. Likewise, without well positioned canines, there may be premature aging and change in facial esthetics.

**Conclusion:**

In order to avoid problems with eruption and positioning of the maxillary canines, early diagnosis is made by analyzing their position and their relationship with the other teeth, and in the three-dimensional context of the maxilla, between 8-10 years of age. Preventive measures can create bone space and direction so that the maxillary canines can occupy their position in the dental arch.

## RESUMO

**Justificativa:**

Os caninos representam esquinas na arcada dentária e são importantes na estética facial, por suportarem o lábio superior e a asa do nariz e influenciarem no sulco nasolabial e no aspecto do envelhecimento facial. Na guia de movimentos de lateralidade, os caninos coordenam a abertura e o fechamento dos dentes, poupando a ATM de movimentos bruscos.

**Discussão:**

Como resultado da falta de erupção ou do posicionamento inadequado do canino superior, pode ocorrer a perda da guia de lateralidade, que se fará no incisivo lateral superior, induzindo lesões de “trauma oclusal”, como a reabsorção apical externa inflamatória. Do mesmo modo, sem os caninos bem posicionados, pode haver envelhecimento precoce e estética facial modificada.

**Conclusão:**

Para não acontecerem problemas de erupção e posicionamento dos caninos superiores, o diagnóstico precoce é feito pela análise da sua posição e da sua relação com os demais dentes e no contexto tridimensional da maxila, entre os 8 e 10 anos de idade. Medidas preventivas podem criar espaço ósseo e direcionamento para que os caninos superiores ocupem a sua posição na arcada dentária.

## INTRODUCTION

John Hunter in the UK denominated the teeth, nearly 300 years ago; except for the canines, which were already named, and they were also known as fangs - especially in wild beasts, which used them to seize their prey, by fixing these fangs in their flesh. The large conical crowns, with long, wide roots, supported in the maxilla by a very dense bone, allowed the animal to shake its neck repeatedly, a strategy used to kill the prey. In humans, canines are outstanding, although they are still smaller than they are in animals.

Canines are the corners of the dental arch. By analogy, street corners in cities are very important in the flow of people, in the logistics of cars and in the esthetics of metropolises. In the mouth, the same occurs with the canines, which guide and determine the paths of food, chewing and the lateral openings of the mouth, sparing and preserving the TMJ. But canines are much more than corners of the arches.

The roots of the maxillary canines and the surrounding bone physically support the wing of the nose and upper lip, without revealing the nasolabial groove, which descends to the corner of the mouth, or commissure. Anatomically, the root and periodontal tissues of the maxillary canine are located below the wing of the nose. The bulging bone of the canine prominence on the buccal surface of the maxilla supports the upper lip, giving the nasolabial fold an appearance of filling.

The wing of the nose and the nasolabial fold are very important in the esthetics of the face. Canines prevent the appearance of premature facial aging, by preserving the nasal shape and volume of the upper lip. In patients without one or both the canines, the role of the canine in esthetics and facial aging is highlighted. The maxillary canines prevent the upper lip from drooping and accentuating the nasolabial fold, thanks to their proximity to the piriform aperture.^1^

## WHERE IS THE CANINE? PREVENTIVE MANAGEMENT

How can the lack of eruption, disturbances in position and even loss of canines be prevented? These frequent problems occur because the canine is the last tooth to occupy its place in the dental arch, with the exception of the third molars.

The lack of space to accommodate the canines changes the shape and volume of the dental arch, and is capable of generating asymmetries expressed in the smile, such as ungraceful laughter. Almost always, the canine that failed to appear is inside the bone, unerupted, or it has erupted and is in an inappropriate position. Many people have a smaller jaw, with lack of space for all the teeth, thus requiring orthodontic and orthopedic corrections performed by specialists. The absence of maxillary and mandibular canines due to partial anodontia is very rare.

The ideal is to know where the maxillary canines are when the patient is between 8-10 years old, after a consultation with the pediatric dentist and/or the orthodontist. Before nine years of age, no permanent tooth will have undergone complete development of its root.^2^

To know the exact position of the maxillary canine, panoramic and periapical radiographs are necessary, as well as tomographic images, including 3D images of the maxilla. At this time, the spatial relationship with the other teeth and its position in the maxilla is established. For other jaw problems, this examination of the jaws and teeth should be done when the patient is six years old.

Two factors contribute to the greater possibility of eruption disorders in maxillary canines:

1) It is the last tooth to position itself in the arch.
2) It is the only tooth that is related to two other teeth to enable its normal eruption to occur, as it is located on top of the deciduous canine and the germ of the permanent premolar.^1^

If the changes are diagnosed at this age, there is a set of procedures that anticipate problems with the canines, and they will occupy their proper position in the dental arch and in the bone that supports the soft tissues, including the upper lip and the wing of the nose. Without presence of the original canines, the middle third of the face is left with a fallen appearance that tends to be noted by an attentive observer. The canine gives the midface an appearance of youthful, fullness and volume.

## OCCLUSAL FUNCTIONS OF THE CANINES

The canines are not only key parts in the esthetics of the face, but they are also fundamental for the proper functioning of the set of teeth and mouth, as we can point out:

1) When we move the mandible to one side, the teeth are guided by the canines and thus lift and separate the maxillary and mandibular posterior teeth, to enable the lateral movement to occur.
2) In this way, the canines coordinate these neuromuscular functions, as they capture the stimuli in their periodontal tissues and, thus, conduct the mandibular movements of closing and laterality, to protect the TMJ.

## WHEN THE LATERAL TEETH ACT AS CANINES, AND THE EIGHT SIGNS OF OCCLUSAL TRAUMA

**Figure 1:**
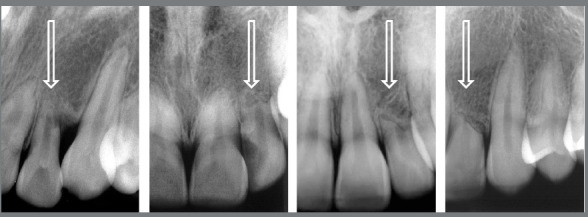
Four maxillary lateral incisors that have served as laterality guide for a period longer than 1 year, which would naturally and properly occur as function of the maxillary canines. The masticatory and occlusal overload induced establishment of the occlusal trauma lesion in its most advanced form, and it is characterized by inflammatory root resorption in plane ( arrows ). Pulp vitality is preserved.

**Figure 2:**
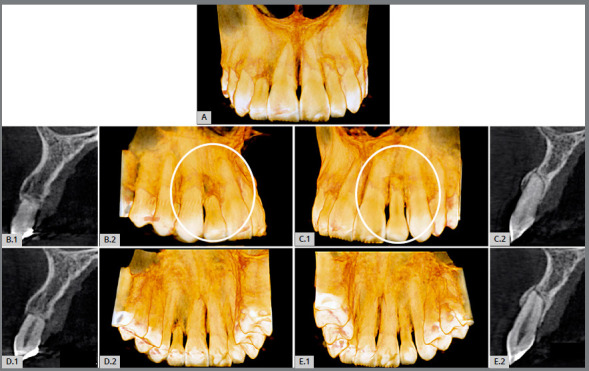
Inflammatory root resorption in lateral maxillary incisors, when they have acted as a laterality guide for a long time; this can occur unilaterally or bilaterally. In this case, the 3D tomographic images highlighted the bilateral resorptions in the vestibular (**A**, **B.2** and **C.1**) and palatal (**D.2** and **E.1**) views, representing two advanced occlusal trauma lesions. In **B.1**, **C.2**, **D.1** and **E.2**, sagittal tomographic images of the lateral incisors can be seen. Analyzing the maxillary canines between 8 -10 years old can prevent this situation (the patient was a 31-year-old woman with a history of previous orthodontic treatment).

Not rarely, healthy maxillary lateral incisors - that is, without caries, without pulp necrosis and without previous history of dental trauma - may be found with external inflammatory external apical resorption unilaterally or bilaterally,^3^ ^,^ ^4^ as in Figures 1 and 2.

In this situation described above, our experience with several cases has almost always led us to indicate this resorption as being part of the lesion or disease called “occlusal trauma”, with late manifestation of months and years of progression and preceded by other manifestations, which we will discuss next. Since the beginning of this “occlusal trauma” lesion, it has been caused by the chewing overload determined on the maxillary lateral incisors in this situation, when these teeth have been used as a guide for the movement of laterality, and not the canines (Figs 1 and 2).

The structure of the maxillary lateral incisors is much more fragile than that of the canines; they are unable to dissipate all the force received and, slowly and individually, the cementoblasts of their apical third become necrotized, thus promoting this resorptive process as the last manifestation of the “occlusal trauma” injury, at all times maintaining pulp vitality.^3^ ^,^ ^4^

The term “injury” means any temporary or permanent anatomical change in our tissues, irrespective of their nature and origin. The term “disease” or illness almost always implies an evolutionary process of a clinical and imaging condition that is repeated when those causes act on the site.

The injury or disease called “occlusal trauma” may be caused by a) occlusal interference; b) chewing overload; c) parafunctional habits, such as bruxism and clenching; and d) active or loose orthodontic retainers. For the “occlusal trauma” injury to become fully established, a prolonged time is required, longer than one year.

The chewing overload can be on one tooth only, as it can occur when the laterality guide function of opening the maxillary and mandibular teeth performed by the canines begins to be performed by the maxillary lateral incisors instead, either unilaterally or bilaterally.

Sequentially and gradually^3^ ^,^ ^4^, the “occlusal trauma” injury establishes itself in the periodontal supporting tissues and shows:

1) radiographic thickening of the lamina dura;
2) irregular enlargement of the periodontal space;
3) increased periapical or lateral bone density;
4) vertical bone loss in the cervical region of the root, with no sign of periodontal pocket or other inflammatory signs.

In the coronal tissues, simultaneously with the previous mentioned signs, the following may appear:

1) the attrition wear facet in the areas of occlusal interference;
2) “V”-shaped gingival recession;
3) presence of abfraction in the cervical enamel.

The last sign of occlusal trauma injury to appear is inflammatory root resorption because the long-term overload will slowly and gradually, focally necrotize cementoblasts in the root surface; and in these bare areas, this will attract the clasts, thus initiating the resorptive process.

## FINAL CONSIDERATIONS

By disseminating the following information, we must make the specialties and population aware of the need to examine the spatial and structural situation of the maxillary and mandibular canines in all children when they are between eight and ten years old. In order to prevent problems in the teeth and jaws, the ideal would also be to evaluate the jaws at the age of about six years.

The methodical and protocol examination of children in the age group between eight to ten years of age would prevent the problems associated with eruptive and space disorders of the maxillary canines. This would lead to great benefits for future adolescents and adults.

The harmonious smile and the health of the dental arch and TMJ, associated with the esthetic aspects of a much more beautiful facial ratio, are highlighted by the canines present in the two corners of the maxillary dental arch. Analyze the faces of celebrities and acquaintances you consider beautiful: you will find that the canine will be there, and this is amazing.

When proposing and justifying preventive treatments for eruptive and space disorders of the maxillary canines, including expanders and dental traction, these points should be explained to patients and their guardians in an illustrated way: their receptivity, understanding and adherence to treatment will be surprising.
